# Identification, Mechanism, and Treatment of Skin Lesions in COVID-19: A Review

**DOI:** 10.3390/v13101916

**Published:** 2021-09-24

**Authors:** Diego Fernández-Lázaro, Manuel Garrosa

**Affiliations:** 1 Department of Cellular Biology, Histology and Pharmacology, Faculty of Health Sciences, University of Valladolid, Campus of Soria, 42004 Soria, Spain; 2 Neurobiology Research Group, Faculty of Medicine, University of Valladolid, 47005 Valladolid, Spain; manuel.garrosa@uva.es; 3 Area of Histology, Faculty of Medicine, Institute of Neurosciences of Castile and Leon (INCYL), University of Valladolid, 47005 Valladolid, Spain

**Keywords:** coronaviruses, COVID-19, SARS-CoV-2, skin lesions, cutaneous manifestations

## Abstract

Coronavirus disease 2019 (COVID-19) is a multisystem disease caused by Severe Acute Respiratory Syndrome Coronavirus 2 (SARS-CoV-2), that primarily causes respiratory symptoms. However, an increasing number of cutaneous manifestations associated with this disease have been reported. The aim of this study is to analyze the scientific literature on cutaneous manifestations associated with SARS-CoV-2 by means of a narrative literature review until June 2021. The search was conducted in the following electronic databases: Medline (PubMed), SciELO, and Cochrane Library Plus. The most common cutaneous manifestations in patients with COVID-19 are vesicular eruptions, petechial/purpuric rashes, acral lesions, liveoid lesions, urticarial rash, and maculopapular-erythematous rash. These manifestations may be the first presenting symptoms of SARS-CoV-2 infection, as is the case with acral lesions, vesicular eruptions, and urticaria. In relation to severity, the presence of liveoid lesions may be associated with a more severe course of the disease. Treatment used for dermatological lesions includes therapy with anticoagulants, corticosteroids, and antihistamines. Knowledge of the dermatologic manifestations associated with SARS-CoV-2 contributes to the diagnosis of COVID-19 in patients with skin lesions associated with respiratory symptoms or in asymptomatic patients. In addition, understanding the dermatologic lesions associated with COVID-19 could be useful to establish a personalized care plan.

## 1. Introduction

Coronaviruses (CoVs) are a large family of zoonotic, single-stranded, enveloped RNA viruses. CoVs can mutate and recombine rapidly, leading to new CoVs. Coronavirus disease 2019 (COVID-19) is a multisystem disease caused by Severe Acute Respiratory Syndrome 2 (SARS-CoV-2), which primarily causes respiratory symptoms [1]. Currently, the world continues to suffer from the COVID-19 pandemic. Its transmission probability has been estimated at the onset of the pandemic at 1.4–2.5 [2] resulting in at least 203,391,279 cases of COVID-19 illness and 4,303,090 deaths have been reported. These numbers continue to rise despite the administration of 4,454,162,834 doses of vaccine administered [3].

SARS-CoV-2 is transmitted mainly through virus-containing respiratory droplets or by contaminated objects [4]. The incubation period varies between 2 and 14 days, with an average of 5 days [5]. The mechanism of SARS-CoV-2 infection depends on the binding of the S protein of the virus to a receptor that is a metallopeptidase called Angiotensin-converting enzyme 2 (ACE2). ACE2 has been identified as the functional receptor for SARS-CoV-2. The localization of ACE2 protein occurs in almost all human organs such as oral and nasal mucosa, nasopharynx, lung, stomach, small intestine, colon, skin, lymph nodes, thymus, bone marrow, spleen, liver, kidney, and brain [6,7]. The ACE2 receptor is present mostly in humans in the epithelia of the lung and small intestine, which could provide potential entry routes for SARS-CoV-2. This epithelial expression, together with the presence of ACE2 in vascular endothelium, also provides a first step in understanding the pathogenesis of the main clinical manifestations of COVID-19 [8,9] (Table 1).

Most confirmed cases of COVID-19 are by detection of viral RNA by real-time reverse transcriptase-polymerase chain reaction (RT-PCR) techniques [10] present with flu-like symptomatology, muscle aches, runny nose, sore throat, gastrointestinal symptoms, and loss of the senses of smell and taste [11]. However, 20% of patients develop severe symptoms associated with respiratory difficulties and pneumonia. In addition, coagulation disorders, septic shock, multiorgan failure, and complications secondary to a systemic inflammatory response are associated with increased mortality [12]. To date, the current availability of drugs to treat SARS-CoV-2 infections, except for dexamethasone, remains limited to supportive treatments such as supplemental oxygen and mechanical support in severe and critical cases [11].

The skin, including mucous membranes, is an organ that frequently presents lesions caused by viral infections (e.g., dengue, Zika, measles, chickenpox, herpes zoster among others) [13,14]. These infections may be primarily localized in the skin or manifest at the cutaneous-mucosal level as part of the general symptomatology [15]. In this sense, several findings have been described in the skin of patients with COVID-19, including morbilliform rash, maculopapular and purpuric lesions, urticaria, acral lesions, chilblains, vesicular eruptions, erythematous rash, millimetric erythematous-purpuric macules on the flexures at the periaxillary level, purple-red papules on the fingers, and male hair loss “androgenic alopecia” [16,17]. The different cutaneous manifestations could be due to two mechanisms: (i) the interaction of the virus with the skin, through the ACE2 receptor of the basal layer of the epidermis located in the membrane of the host cell; (ii) hyperactive immune responses, by complement activation or by microvascular injury [18,19,20].

At the beginning of the COVID-19 pandemic, the prevalence of skin lesions was 0.2%, but the types of lesions or their characteristics were not described [21]. Over time, as the COVID-19 pandemic progressed, the frequency of dermatological changes reported varied between 5% and 20% [22]. Thus, the incidence of cutaneous manifestations related to COVID-19 has progressively increased, in parallel with the spread of the global SARS-CoV-2. However, the time of onset is difficult to determine [22,23].

The occurrence of SARS-CoV-2 viral infection induces cutaneous manifestations, whose dermatological lesions need to be sought because these cutaneous signs are heterogeneous, frequent, and varied [24], and of interest for the diagnosis since they can help in the early detection of COVID-19 infection in individuals who do not have other symptoms of SARS-CoV-2 infection and might otherwise go undetected [24]. Thus, skin lesions that precede general symptoms or are even the only sign of positive COVID-19 could serve as early indicators of disease or as indicators of asymptomatic carriers of the virus. In addition, some types of lesions precede the development of the more typical COVID-19 symptomatology and there is even evidence that some types of lesions lead to a worse prognosis and evolution of the disease [25]. This would make it easier therefore to predict a more serious or severe course of SARS-CoV-2 infection. Cutaneous symptoms occurring late in the course of infection or even after resolution of the primary symptoms of COVID-19 do not imply SARS-CoV-2 replication or likelihood of contagion [24,25].

The growing number of case reports and clinical series that have recently been published have described a wide spectrum of cutaneous manifestations associated with COVID-19. These cutaneous presentations could be directly related to the virus or complications of the infection [26]. In addition, they could also be due to side effects of the drugs used or that SARS-CoV-2 may not be the cause [20]. For these reasons, finding out the possible relationship between COVID-19 and cutaneous manifestations may help to better understand the pathogenesis of the disease and the adoption of infection control policies. This study presents a literature review of the main cutaneous manifestations of patients with SARS-CoV-2 infection reported so far. Subsequently, several pathophysiological mechanisms that may explain the cutaneous manifestations, as well as the treatment of each of them, are described.

## 2. Material and Methods

### 2.1. Search Strategy

The present study is a narrative bibliographic review carried out between February and September 2021 that sought to analyze the updated literature on the cutaneous manifestations associated with SARS-CoV-2. The bibliographic search was conducted in the following electronic databases: Medline (PubMed), SciELO, and Cochrane Library Plus. Several terms (Mesh) were used as keywords for the search: Coronavirus, COVID-19, Skin Manifestations, Dermatologic, Exanthema, Maculopapular, Urticaria, Chilblain, Vascular lesions, Vesicular eruptions, Treatment, Hair, Nails, Mucous Membrane, SARS-CoV-2 variants, Vaccine and Long Covid in addition to the Boolean operators “AND” and “OR” as a search nexus. Using the snowball methodology, another 2 articles were selected.

After searching the articles in the databases, the search titles were cross-checked to identify duplicates and potential publications to add. After reading the abstract, a full text review of the selected articles was performed.

### 2.2. Inclusion and Exclusion Criteria

The following inclusion criteria were applied to select articles: (1) Full text access; (2) being a review, clinical trial, observational study, case report/study; (3) identifying skin manifestations related to SARS-CoV-2: maculopapular rash, urticaria, acral lesions, petechiae/purpura, liveoid lesions, vesicular eruptions; (4) papers whose publication date was between 2020 and 2021; (5) languages were restricted to English, German, French, Italian, Spanish, and Portuguese. Regarding exclusion criteria, the criteria applied were: (1) Publications not related to cutaneous manifestations of the new coronavirus that did not differentiate prevalence, distribution, causative mechanism, and/or treatment; (2) duplicate papers; (3) articles published before 2020.

## 3. Results and Discussion

The first description of patients with cutaneous manifestations associated with SARS-CoV-2 was reported in Italy [27]. Recalcati [27] reported that 20% of patients in his sample developed cutaneous manifestations such as erythematous rash, generalized urticaria, and varicella-like vesicles. Other studies [19,22,23,25] classified dermatological lesions related to SARS-CoV-2 infection according to their morphology: vesicular rashes, petechial/purpuric rashes, acral lesions, liveoid lesions, urticarial and maculopapular rashes. Sameni et al. [20] analyzed the frequency of skin manifestations associated with SARS-CoV-2 according to case reports/series, and among the types of comorbidities recorded in the studies, obesity, diabetes (7.4%), and hypertension (5.5%) were the most common comorbidities reported in patients with dermatologic lesions associated with COVID-19.

González et al. [28] proposed a pathophysiological hypothesis based on the interpretation of various clinical manifestations related to COVID-19, dividing these expressions into two categories: (i) Those manifestations that are mainly based on the direct viral cytopathic effect on cells of the organism such as keratinocytes, including in this group maculopapular, urticaria and chickenpox-like eruptions; (ii) those manifestations due to changes in specific white blood cells, T lymphocytes, and macrophages, such as secondary infection with uncontrolled overexpression of cytokines (stage of cytokine storm). In turn, the latter group will be subdivided into: (a) Dermatological manifestations with features such as macrophage activation syndrome including acral ischemia, gangrene, petechiae/purpura; (b) liveoid lesions, which are associated with high patient morbidity and mortality; and (c) cutaneous manifestations due to activation of an early response to type I interferon (IFN-1), including chilblain-like lesions [28].

**Figure 1:**
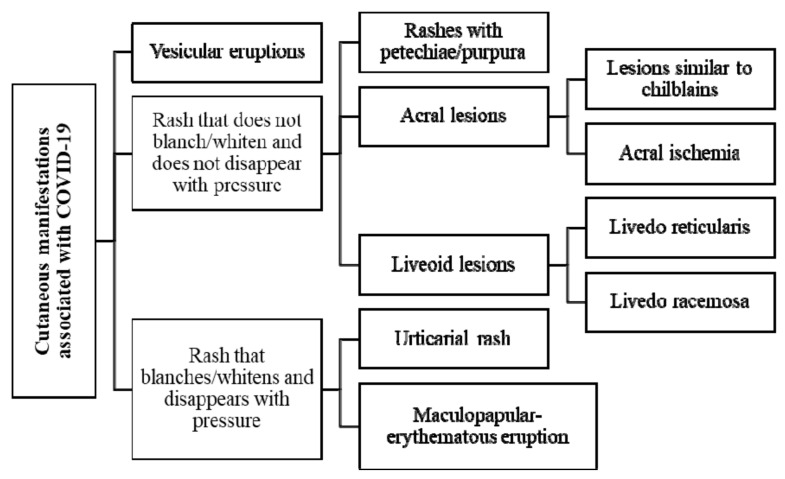
COVID-19 and skin manifestations.

For a better understanding of the dermatologic lesions related to SARS-CoV-2 infection, due to the heterogeneity of the cutaneous manifestations (Figure 1), the different types of lesions will be analyzed according to patterns of vesicular eruptions, scabs, and erosions; lesions that do not disappear with pressure such as petechiae/purpura, acral lesions, liveoid lesions; lesions that disappear with pressure such as urticarial and maculopapular-erythematous rash; as well as their triggering mechanism and the proposed treatment for each one of them (Table 2).

### 3.1. Vesicular Eruptions

**Figure 2:**
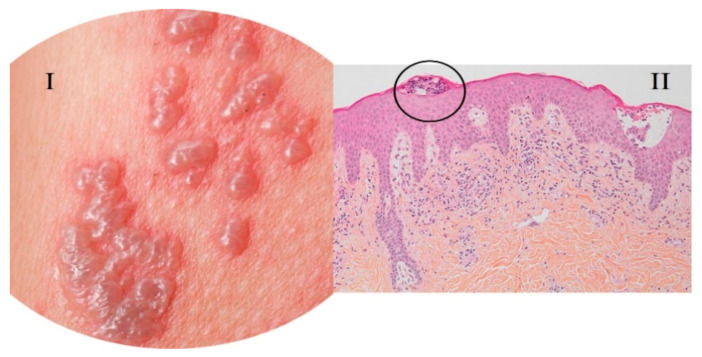
Vesicular lesions associated with COVID-19. (**I**) Vesicular eruptions associated with COVID-19; (**II**) skin biopsy with spongiotic vesicular eruption. HE × 10. Arias-Arguello A. (2020) [32].

Vesicular eruptions are papules of less than 1 cm commonly transparent and filled with liquid under the epidermis [22] (Figure 2). In COVID-19, the prevalence of vesicular lesions is 15%, identifying a diffuse pattern in 75% and a localized pattern in 25% of patients [29]. Perna et al. [30] described a prevalence of 12.5%, while according to a series of cases analyzed by Singh et al. [22]—this percentage varies between 3.7% and 15%. These lesions occur more frequently in adults, most of them male (72.7%). The average age is between 50 and 70 years [28,31], although younger people, including children, can be affected [24].

Vesicular lesions are mostly located on the trunk and to a lesser extent on the extremities: diffuse pattern eruptions tend to affect more than one body area, palms of the hands, and soles of the feet; localized pattern lesions tend to affect the central area of the body [29]. Sameni et al. [20] have identified cutaneous manifestations such as exanthema with lesions similar to chickenpox. These can occur on the hands, feet, and trunk, and can sometimes be accompanied by pruritus and even pain. We differentiate vesicular eruptions from varicella in that, unlike varicella, these vesicles are monomorphic. The onset of vesicular lesions is usually later than that of SARS-CoV-2 disease symptoms, with a latency time of 14 days occurring more frequently in middle-aged patients [20]. However, according to other studies [30,33], these lesions appeared earlier than other clinical manifestations of COVID-19 in 72.5% of cases and were associated with a medium severity of SARS-CoV-2 infection. In this way, Jindal et al. [31] reported that vesicular lesions occur in the early stages of COVID-19, even before the onset of fever and cough, and last about 10 days. In relation to other symptoms of COVID-19, most patients with these manifestations present a full clinical picture of the disease with systemic and respiratory manifestations. Regarding the severity of COVID-19 in patients with these cutaneous vesicular manifestations, Fernandez-Nieto et al. [29] described that 58.3% presented mild symptoms of the disease without requiring hospitalization and 41.7% developed pneumonia. Among them, only 4.2% required admission to the intensive care unit (ICU) and all patients survived. However, Mawhirt et al. [34] reported that the mortality rate of these patients was 13.6%. Thus, vesicular lesions in the context of SARS-CoV-2 infection are mainly observed in the early stages of the disease (active phase) and occur in mild to moderate forms of the disease in adults or children. These cutaneous manifestations may occur before the onset of other symptoms of SARS-CoV-2 infection. In addition, vesicular eruptions have been described as “specific cutaneous manifestations” related to COVID-19, therefore, their identification can be of great help in the diagnosis of the disease [23]. Differential diagnosis should include herpes simplex manifestations and herpes zoster (reactivations of herpes simplex and herpes zoster have been described in patients with COVID-19), erythema multiforme, and autoimmune blistering diseases [33,35].

No treatment is available for the vesicular eruptions associated with SARS-CoV-2; these lesions resolve within a few days, a period of about one week, and without scarring. Because of this, the treatment would be an active observation of the patient’s condition without administering treatment, unless other symptoms appear, or the patient’s condition changes, which is what we would call expectant management [24].

### 3.2. Lesions with Vascular Component

SARS-CoV-2 has been associated with alterations in coagulation and vascular damage [36]. Lesions with a vascular component related to SARS-CoV-2 infection were the second most frequent, after maculopapular eruptions. By applying light pressure to the lesion area, we can observe whether the lesion pales or disappears after pressing for a few seconds [37]. These lesions with a vascular component may be associated with common symptoms of COVID-19 such as fever, dry cough, and myalgia [33]. Lesions with a vascular component that do not disappear with pressure can be differentiated into: (i) Rashes with petechiae/purpura; (ii) acral lesions; (iii) liveoid lesions

#### 3.2.1. Rashes with Petechiae/Purpura

**Figure 3:**
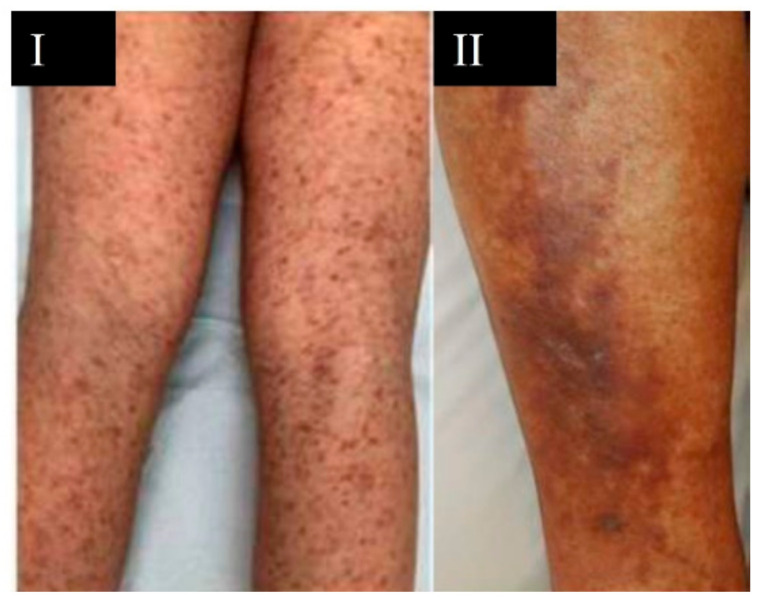
Purpura associated with COVID-19. (**I**) Purpuric lesions on the lower extremities; (**II**) purpuric plaques on the leg. García-Molina C. (2020) [38].

Eruptions composed of red macules that do not blanch and that are distributed in a generalized manner can be indicators of eruptions with petechiae/purpura [36]. Petechiae are those spots with a diameter of less than 2–4 mm and purpura lesions measuring between 4 and 10 mm in diameter [22] (Figure 3).

The prevalence of petechiae/purpura rashes is 4% in COVID-19 patients who did not require hospitalization [35], while in hospitalized patients the prevalence of purpura was 25.7% [39]. These eruptions are frequent in middle-aged and elderly adults and without significant differences between sexes [39]. The location of petechial eruptions can be diffuse or on the extremities, while purpura lesions are located on the legs and buttocks [35] or in a generalized manner, localized in intertriginous regions or with an acral distribution [40]. Petechiae and generalized purpura usually appear in the most severe cases of SARS-CoV-2 infection. According to a study by Rekhtman et al. [39], 66.7% of patients with purpura were mechanically ventilated and Freeman et al. [23] described that 100% of patients with purpura were hospitalized and within them, 82% had acute respiratory distress syndrome (ARDS). These lesions have a high mortality rate [23]. Rashes with petechiae/purpura secondary to small subdermal hemorrhages could be considered as a cutaneous manifestation related to COVID-19 and appear at any time during the course of the disease [35,40].

The pathophysiological mechanism of purpura lesions could be due to severe microvascular injury mediated by complement activation [40] or by cytokine storm [28]. The petechial exanthema associated with COVID-19 may be causing thrombocytopenia, mimicking dengue disease in endemic areas. For a correct differential diagnosis, patients should be evaluated for respiratory symptoms, fever, and petechial lesions [41]. Treatment of these cutaneous eruptions includes therapy with topical corticosteroids in mild cases and systemic corticosteroids in more severe or generalized involvement [42].

#### 3.2.2. Acral Lesions

**Figure 4:**
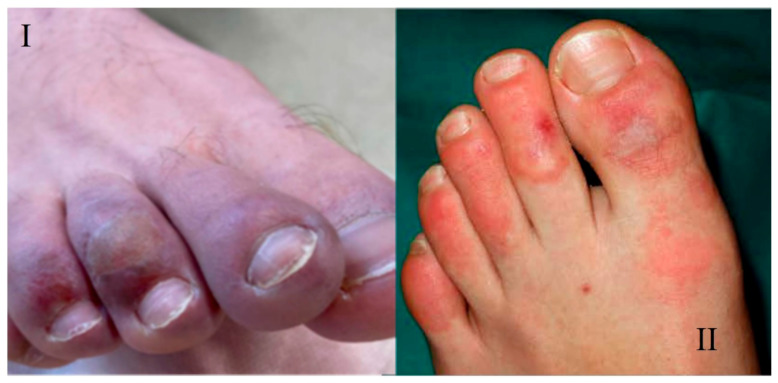
COVID fingers. (**I**) COVID-19 toes; (**II**) mixed pattern composed of dactyliotis and macula papules purpura. Arias-Arguello A. (2020) [32].

The skin lesions that do not blanch and are distributed in the distal areas of the extremities can be indicators of acral lesions such as chilblain-like or pernio-like lesions and acral ischemia [37]. These lesions, related to COVID-19, present without a history of exposure to cold and the main affected parts are the fingers and toes. Morphologically, they are characterized by erythematous-violaceous papules and macules, with edema and possible blistering evolution; they may also be accompanied by pain and pruritus [22]. On the other hand, an acral arrangement of violaceous lesions that do not pale is indicative of acral ischemia [40]. Terms used to describe acral eruptions associated with SARS-CoV-2 include: acroischemia, pernio-like or perniosiform eruptions, pseudo-scabies, and scabies-like eruptions. These lesions, due to their incidence and association with SARS-CoV-2 infection, are referred to as “COVID fingers” [29] (Figure 4).

Perniosiform lesions have a variable prevalence: 14–72% [22], 15% [31], and 19% [33]. These lesions are more frequent in adolescents and young adults, with involvement of the feet and fingers in 71% of the cases and in the same proportion in men and women [20]. In this way, Cappel et al. [43] showed that 63% of patients with cutaneous manifestations presented lesions similar to chilblains and 94% of patients were located on the feet. The appearance of chilblain-like lesions follows the onset of systemic symptoms of COVID-19, with the mean duration of these lesions being around 13 days [20,22]. These lesions were associated with less severe disease in terms of hospital admission, pneumonia, ICU admission, or morbidity and mortality [20,22]. According to Freeman et al. [44], 16% of patients with pernio-type lesions were hospitalized, compared with 35% for all other dermatologic manifestations of SARS-CoV-2 associated disease. In a study by Cappel et al. [43], 98% of patients with these lesions received only outpatient care, 55% had no symptoms of the disease, and 45% developed respiratory manifestations [33]. The clinical finding of chilblain-like or pernio-like lesions is an unusual manifestation of COVID-19, suggesting it to be an effective host antiviral response by the host [19]. These lesions appear more frequently during the late evolution of the disease, so they could be useful as epidemiological markers or as a possible sign of COVID-19 in patients with scarce and/or poorly expressive symptomatology [22].

The causes attributed to COVID-19-associated chilblain-like lesions raise a hypothesis that SARS-CoV-2 causes coagulation disorders (increased d-dimer and fibrinogen degradation products), thrombosis, generalized endothelial infection (endothelial damage) [43], and dysregulation of immune pathways (early IFN-1 response) [19]. In addition, chilblains could be a paraviral rash associated with COVID-19 [45]. Although these lesions tend to resolve spontaneously in 1 to 4 weeks, topical corticosteroid therapy alone or in combination with topical antibiotics has been proposed as a treatment for chilblain-like lesions [42].

The reported prevalence of acral ischemia is 6% [33]. These lesions are frequent in elderly patients and in those with severe SARS-CoV-2 disease [33]. Acral ischemia manifests as necrosis with localization in fingers and toes [46]. Approximately 80% of patients with acral ischemia/necrosis required mechanical ventilation [39]. The mortality rate of patients with acral lesions is 71% [34]. These lesions are considered secondary to a context of vascular micro-occlusion and acral ischemia due to a general deterioration of the patient and/or coagulation disorders attributed to COVID-19. Some patients with acral ischemia were diagnosed with disseminated intravascular coagulation (DIC) [35]. Therefore, treatment of this cutaneous manifestation includes anticoagulant therapy [40].

#### 3.2.3. Liveoid Lesions

**Figure 5:**
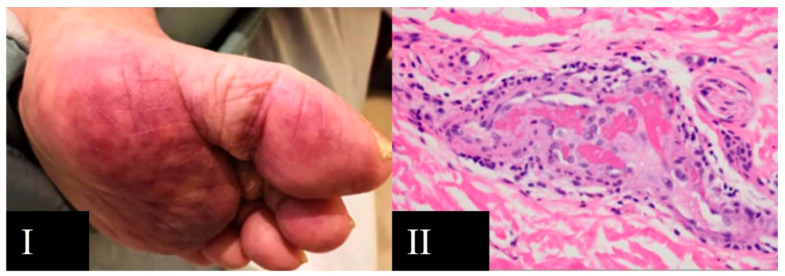
Livedo reticularis associated with COVID-19. (**I**) Livedo reticularis in the plantar region; (**II**) skin biopsy showing pauci-inflammatory thrombogenic vasculopathy (HE × 400). Arias Arguello A. (2020) [32].

Genovese et al. [27] define liveoid lesions as “lesions with a reticulated pattern of slow blood flow, with consequent blood desaturation and bluish skin discoloration”. An incidence of 6% is reported during the first peak of the epidemic in Spain in April 2020 [33]. Liveoid lesions are common in older or elderly adults with more severe SARS-CoV-2 infections and with no significant differences between sexes [46]. Two types of liveoid lesions can be differentiated: (i) Livedo reticularis or necrosis, which is a cutaneous manifestation occurring transiently or persistently with a reticulated vascular pattern of reddish and bluish skin coloration (Figure 5); (ii) livedo racemosa, which is a permanent manifestation with a pattern of discontinuous circles and with the appearance of a more widespread pattern on the body [22]. The prevalence of livedo reticularis is 3.5% and livedo racemosa is 0.6% [35].

The Livedo reticularis is mostly located on the dorsal hands and feet, forearms and antecubital fossa, chest, and legs [46]. Liveoid rashes develop at the same time as other systemic symptoms of COVID-19 and with an average duration of 9 days. Livedo reticularis lesions can occur at any time of the disease, but their progression to papulonecrotic cutaneous vasculitis could indicate complications leading to vascular occlusion, coinciding with an increase in the severity of the disease [46]. Livedo reticularis lesions have been associated with clinical manifestations such as hematuria and possible renal damage, so they are considered as the pattern most associated with mortality, especially in older adult patients, with associated comorbidities and in those with severe forms of the disease [18]. Liveoid lesions, along with purpura lesions, have one of the highest mortality rates among patients with cutaneous manifestations associated with COVID-19, at approximately 10% of SARS-CoV-2 infected patients.

**Figure 6:**
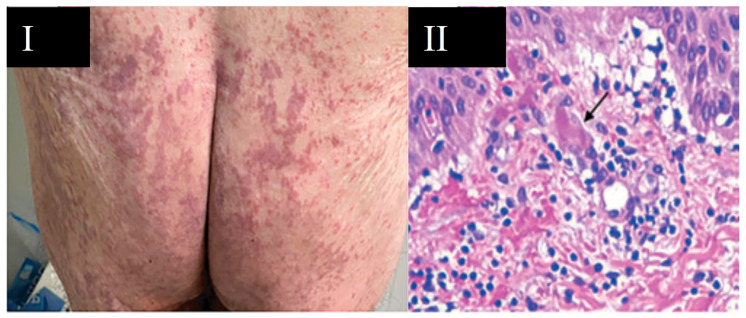
Vascular damage by COVID-19. (**I**) Livedoid lesions or necrosis pattern as a consequence of a vasculopathy; (**II**) skin biopsy with focal thrombosis (arrow) in the papillary dermis capillaries and extravasation of hematomas (HE × 100). Singh H. et al. (2021) [22].

The cause of leveoid lesions is probably due to hypercoagulability (microthrombi), neurological and immunological alterations [46]. The livedo reticularis or necrosis pattern presents a vascular pattern due to alterations in coagulation and vascular damage by COVID-19 (Figure 6). These states induce the overproduction of interleukin 6 (IL-6), which produces vascular thrombosis through its effects on platelet aggregation and activation or on angiotensin II regulation [28]. Moreover, the replication of SARS-CoV-2 within cells induces cell damage with the release of proinflammatory cytokines and activation of the complement cascade. This response allows for the recruitment of leukocytes, lymphocyte proliferation, release of interferon-gamma (IFN-γ), interleukins (IL), especially IL-6, ferritin, and tumor necrosis factor-alpha (TNF-α) [47,48]. This has been related to macrophage activation and consequent macrophage activation syndrome with an uncontrolled expression of cytokines and release of the plasminogen activating factor, this being a possible mechanism that would explain the elevation of d-dimer and thrombotic episodes that can manifest in the skin [48,49].

In addition, the mechanisms of type III hypersensitivity reactions or immunocomplex-mediated reactions to viral antigens which can precipitate and accumulate inside the vessels, are also part of the main processes leading to vascular injury [28]. Vasculitis at the skin level is characterized by hyperemic or violaceous livedoid lesions with a net-like appearance. These lesions may lead to necrosis of the overlying epidermis and the mechanisms involved in this microvascular dysfunction may include direct action of the SARS-CoV-2 on endothelial cells together with an altered immune response [25,46].

The usual treatment of non-severe livedoid lesions includes anticoagulant therapy and expectant management [40]. Other authors reported livedoid lesions as a transient manifestation in some COVID-19 patients who did not require specific treatment [33,35].

### 3.3. Urticarial Eruption

**Figure 7:**
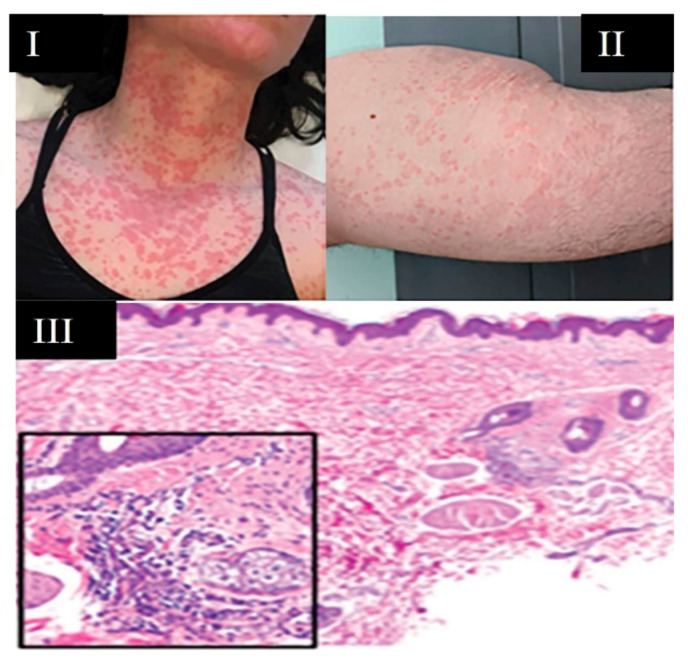
Urticarial Rash. (**I**) Urticarial rash, similar to hives, appearing on patient’s neck and chest; (**II**) urticarial lesions dispersed along buttocks and proximal lower extremity (thigh); (**III**) urticarial pattern with mild edema, perivascular inflammation, and dilated vessels in the upper dermis. Inset: vessels filled with neutrophils and mixed perivascular inflammation. Singh H. et al. (2021) [22].

Palisading/blanching lesions are identified lesions that typically present as urticaria or angioedema and are commonly associated with pruritus [37] (Figure 7). Histamine-mediated angioedema may be accompanied by urticaria or present alone, representing deeper dermal edema [27]. Urticaria is one of the most frequently reported cutaneous manifestations in patients with SARS-CoV-2 infection [50]. In addition, itchy red patches on the hands and feet have been observed in patients with COVID-19 as a consequence of inflammatory processes in the skin [20]. The prevalence of urticaria with respect to all dermatological manifestations associated with COVID-19 is variable, ranging between 19% [33], 16.7% [51], 7–40% [22], and 9% [36], depending on the general condition of the patient. Regarding the prevalence by sex (64% in women and 36% in men), urticarial rash is more frequent in middle-aged people between 45 and 65 years [50,51].

The urticarial eruption was distributed mainly on the trunk or in a generalized manner, and to a lesser extent in facial and/or acral areas [22]. In 55% of cases, urticarial lesions occurred before or at the same time as pseudo-flu symptoms of COVID-19 and with improvement or disappearance in less than a week; in 10% of cases, the onset of urticaria manifested before systemic symptoms of COVID-19 [50,51]. Genovese et al. [24] associated the presence of urticarial eruptions with other symptoms such as anosmia, ageusia, chills, and dizziness. Patients with urticaria presented cough (59–66%), dyspnea (40–45%), and fever (70–80%) [22,31]. Approximately 90% of patients were treated on an outpatient basis [50] and only 11% required ICU admission [51] with low mortality rates [30,31]. Patients with urticarial lesions may develop associated complications such as angioedema [50,51].

Most urticarial lesions may not be very useful for the diagnosis of COVID-19, because the pathways are unclear either these urticarial eruptions represent a manifestation of SARS-CoV-2 itself by direct mast cell degranulation [12] or are induced by drugs associated with COVID-19 treatment [22,31]. Urticaria has been described as a side effect of many drugs used for the treatment of COVID-19, such as chloroquine, hydroxychloroquine, and lopinavir/ritonavir [52], albeit some patients’ urticaria improved without discontinuation of treatment against SARS-CoV-2 infection [42,50]. Treatment of these lesions includes low-dose systemic corticosteroid therapy combined with antihistamines, not only to control urticaria but also to improve morbidity and mortality from SARS-CoV-2 disease. The administration of corticoids requires individual guidelines, and the duration should be as short as possible until the suppression of symptoms [42].

### 3.4. Maculopapular-Erythematous Rashes

**Figure 8:**
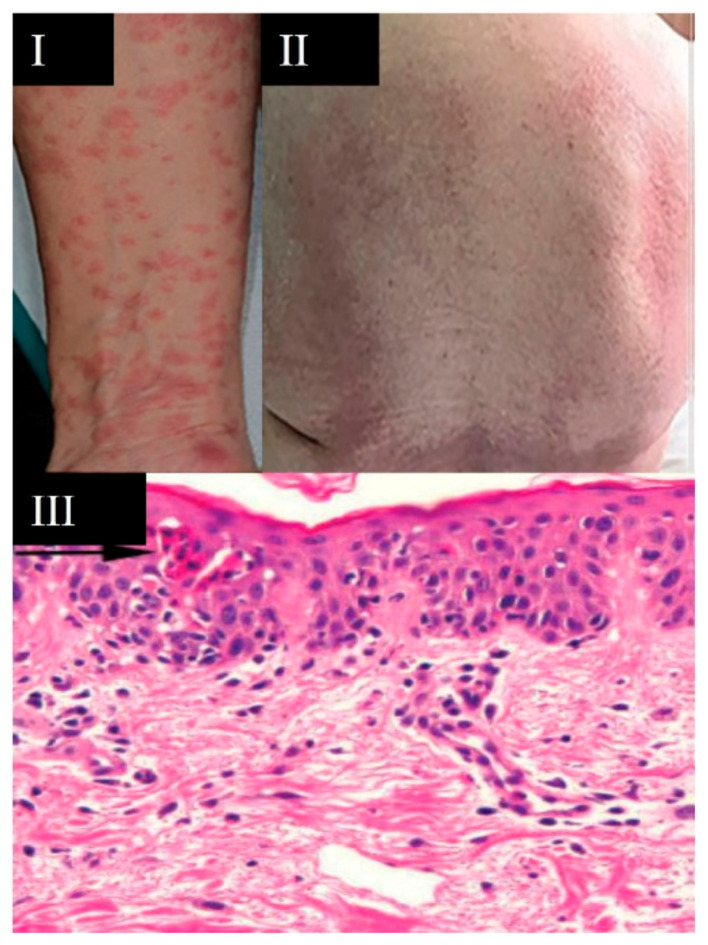
Maculopapular-erythematous rash. (**I**) Maculopapular lesions described as small plaques after fusion of the lesions; (**II**) maculopapular rash appearing on the posterior trunk; (**III**) skin biopsy showing clusters of apoptotic keratinocytes in the epidermis (arrow). Singh H. et al. (2021) (**I**,**II**) [22] and Arias Arguello A. (2020) (**III**) [32].

**Figure 9:**
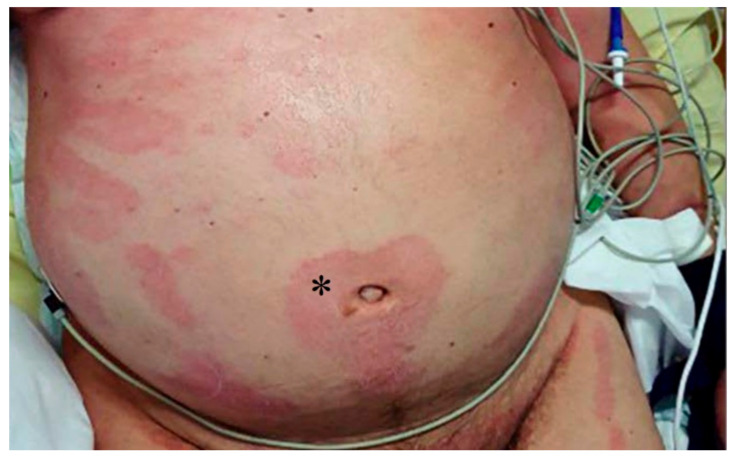
Papule-squamous eruption. * Papule-squamous eruption in abdomen. Sanchez J. et al. (2020) [53].

Maculopapular-erythematous rashes are characterized by an erythematous skin rash, flat erythematous macules, and/or raised papules. Occasionally these rashes may be accompanied by a petechial component or with macules or more extensive areas of purpuric appearance [31,40]. Maculopapular-erythematous eruptions (Figure 8) have the highest prevalence (40–70%) of the cutaneous manifestations associated with SARS-CoV-2 [22,31,35], including (a) erythematous eruptions 38.5%, (b) maculopapular eruptions 18.3%, (c) macular erythema 6.8%, and (d) papulo-squamous eruption (2%) (Figure 9).

Maculopapular-erythematous rashes have been identified in elderly patients and with a localization mainly on the trunk or in a generalized manner, and without significant differences between sexes [31]. Maculopapular-erythematous rashes may be accompanied by pruritus and even pain. Most of them appear after the onset of other symptoms of COVID-19, with a manifestation time of about 20–25 days and an average duration of lesions of 9 days. Catalá et al. [54] described that these eruptions appeared concomitant to other systemic manifestations of COVID-19 in 62% and late stage in 34.1%. The occurrence of this cutaneous manifestation is associated with severe disease and a mortality rate of 2% [33].

Moreover, seven maculopapular patterns are identified: morbilliform, other maculopapular eruptions, purpuric, similar to erythema multiforme, pityriasis rosea, as persistent raised erythema, and perifollicular [54]. Catalá et al. [54] reported that maculopapular rashes are associated with other symptoms of COVID-19: fever (88%), cough (76%), dyspnea (72%), asthenia (62%), nausea/vomiting (30%), headache (29%), and anosmia/ageusia (23%). Hospital admission for pneumonia was very frequent in this type of eruption. Approximately half of the patients with maculopapular eruptions required hospital admission for pneumonia and 2.8% of the cases required admission to the ICU [25]. In relation to the different patterns, hospitalization was required in 80% of morbilliform patterns and in 76.5% of cases of erythema multiforme. In addition, mechanical ventilation was required in about 20% and admission to the ICU in about 12% of cases in this type of pattern [54].

Possible causes of these rashes include immune response to SARS-CoV-2, as the rash often coincides with episodes of fever or other symptoms of COVID-19 [12]. In addition, the direct cytopathic effect of SARS-CoV-2 on keratinocytes and reactivation of human herpesvirus 6 by COVID-19 may explain the presentation of this lesion pattern [28]. Maculopapular exanthema is also typical of other viral infections such as dengue, zika, and measles, mediated by an overproduction of cytokines. In addition, it can be observed as a cutaneous manifestation of allergic reactions to drugs. Therefore, a complete anamnesis (physical examination; previous drug exposure) and the performance of a confirmatory RT-PCR test would be necessary for the diagnosis of SARS-CoV-2 infection versus other endemic viruses [55].

Treatment of maculopapular-erythematous rashes varies according to the severity of the clinical picture, with topical corticosteroid therapy in mild cases and skin hydration and systemic corticosteroids in case of generalized or severe rash [42].

### 3.5. New SARS-CoV-2 Variants and Skin Manifestations

Surprisingly, information on cutaneous manifestations associated with new SARS-CoV-2 variants is very limited [56]. Perhaps, the acquired knowledge of the cutaneous manifestations associated with COVID-19 has resulted in a reduction in the number of case reports. In fact, manuscripts that are accepted in journals are larger cohort studies and/or based on clinical registries of COVID-19 [57]. On the other hand, new SARS-CoV-2 mutations and variants may have resulted in fewer cutaneous manifestations, possibly due to reduced viral cutaneous tropism or by inducing different immune responses [57]. Nonetheless, further studies are needed to confirm this as well as the influence of vaccination.

The delta variant (∆) secretes more nasal discharge and mucus than in the original SARS-CoV-2 variant. The main difference between the ∆ variant and other variants is that rare symptoms, such as severe intestinal disorders and deafness, have been detected in association with the ∆ variant. The differential cutaneous manifestations of the ∆ variant with other variants is gangrene [58]. This could be related to the greater involvement of the vascular system caused by this ∆ variant, which could suggest a higher incidence in lesions of vascular components such as petechiae/purpura, acral lesions, and liveoids [59].

### 3.6. Skin Lesions in Vaccinated Patients

#### 3.6.1. Infected by SARS-CoV-2

The vaccines currently available against the new coronavirus are not “sterilizing”, i.e., vaccines are not designed to prevent replication of SARS-CoV-2 in the mucosa of the upper respiratory tract. Thus, a vaccinated person can become infected and, although they normally do not develop severe COVID-19 because their viral load is greatly reduced, patients may have mild symptomatology. It is to be expected that the fewer viruses a person carries, the fewer signs and/or symptoms [60]. Thus, the cutaneous manifestations of vaccinated patients infected with SARS-CoV-2 could be those early manifestations of SARS-CoV-2 infection and of a mild nature, such as vesicular rash lesions and urticaria [30]. In addition, these cutaneous manifestations of vaccinated patients infected with SARS-CoV-2 could be used as identification signs in asymptomatic patients [35]. However, this aspect needs to be further studied as vaccination progresses worldwide.

#### 3.6.2. Vaccinated without Infection

On the other hand, most of the adverse skin manifestations expected after vaccination against COVID-19 are mild and are related to the presence of acute signs such as erythema and local edema in the area surrounding the inoculation; these are not usually of clinical concern since they are common to other vaccines, and usually persist for a maximum of three days until their complete resolution without health intervention [61].

### 3.7. Abnormalities of Mucous Membranes, Hair and Nails Associated with SARS-CoV-2 Infection

#### 3.7.1. Mucous Membranes

The oral cavity can be altered by COVID-19 disease. Lingual edema with transient U-shaped lingual papillitis or glossitis with patchy epilation are the most frequently encountered signs, as well as a burning sensation in the oral cavity or a burning mouth. Other oral manifestations that may be associated with COVID-19 are mucositis with or without aphthous ulcers or enanthema. All can be key signs for a diagnosis of COVID-19 [62].

In a temporary field hospital set up during the peak of the pandemic (March–May 2020) in Madrid (Spain) to care for COVID-19 patients with mild to moderate pneumonia, 78 patients (25.65%) were found to have oral cavity mucosal alterations, including transient tongue papillitis in 35 patients (11.5%), glossitis with patchy epilation in 12 patients (3.9%), and aphthous stomatitis or mucositis in another 12 (3.9%). Burning mouth or lingual burning sensation was registered in 16 patients (5.3%) and most of them presented taste alterations (dysgeusia) [63].

#### 3.7.2. Hair

If we have learned anything from the COVID-19 pandemic is that the unexpected is to be expected. However, the hair loss that many patients, who have had SARS-CoV-2 infection, develop may not be so unexpected because temporary hair loss is normal after fever, surgery, or illness, fever being a common symptom of COVID-19 [36]. The multi-organ symptoms triggered by SARS-CoV-2 infection cause a major impact on the body; the body goes into lockdown mode and only concentrates on essential functions [36]. Hair growth is not as essential as other functions, so patients end up with hair loss [64].

In addition, this alopecia can also be increased by the physical and emotional stress accompanying a case of SARS-CoV-2 infection or by the current situation of restrictions and changes of life habits adapted to new normality [56]. Therefore, the stress accompanying COVID-19 can lead to a reversible hair loss condition called telogen effluvium and occurs a few weeks after the acute COVID-19 process [65].

#### 3.7.3. Nails

Nails, as for hair, can reflect the state of health of the patient in recent months, as the nails of the hands grow on average about 2 mm per month [64]. Cases are emerging in which, after infection with SARS-CoV-2, certain changes have been observed in the fingernails and toenails, which are popularly known as “COVID-19 nails” [66]. Although, dermatologically speaking, one could not speak of COVID-19 nails as such, but rather that there are manifestations in the nails because of COVID-19. These are horizontal spots and indentations on the surface of the nails that manifest themselves a month or two months after SARS-CoV-2 infection [56]. These lesions appear as small grooves and the longer the involvement lasts, the wider the band becomes, albeit these alterations in the nails can also be triggered later after any important infectious process or in any disease besides COVID-19 [66,67]. In this sense, Beau’s lines can appear, in addition to COVID-19 [68], in diseases such as pneumonia or hand, foot, and mouth disease, and are due to the fact that the body is wise and prefers to focus all its functionality on the vital organs, rather than in the secondary ones, such as the nails [64].

There are some nail manifestations that have only been reported in people infected with SARS-CoV-2 as follows: (i) Red crescent: red bands bordering the lunula, occurring in all nails. This has been seen in a small group of patients with COVID-19 and is thought to be related to pernio-like lesions. This could occur because SARS-CoV-2 reaches the distal zone and plugs the most superficial capillaries of the skin leading to this type of red lesions [69,70]; (ii) orange transverse lines: in patients in the acute phase of COVID-19, horizontal lines have been visualized, showing a stronger orange color and a red color of the nail bed, whereas in healthy patients the tone is usually pink [66,67].

### 3.8. The Manifestation of Skin Rash as a Symptom in Long-COVID Patients

The long-COVID (LC) patient develops a multiorgan symptomatic complex that remains after the acute phase of the disease, after 4 or even 12 weeks, with symptoms persisting over time and cases being considered not to have a disease-free period, although the clinical picture is characteristically fluctuating [71]. There are multiple dermatological manifestations in those afflicted by LC, and in many cases, they are reminiscent of dermal lesions caused by other viruses such as Parvovirus B19 [72]. In this regard, morbilliform rashes had a median duration of 7 days (RIQ: 5 to 10 days), while urticarial eruptions persisted for a median of 4 days (RIQ: 2 to 10 days) among patients with laboratory-confirmed COVID-19, with a maximum duration of 28 days. The median duration of papulosquamous eruptions was 20 days (RIQ: 14 to 28) in patients with laboratory-confirmed COVID-19, the rash persisting for 70 days in one of these patients. Erythema pernio (chilblains) had a median duration of 15 days (RIQ: 10 to 30 days) in patients with presumptive diagnosis of COVID-19, and 12 days (RIQ: 7 to 23) in laboratory-confirmed cases. Seven out of 103 patients (6.8%) had such lesions for more than 60 days, the infection being laboratory confirmed in two of them [73].

Urticarial and morbilliform eruptions are usually of short duration, whereas papulosquamous eruptions, and especially erythema pernio, tend to persist for longer periods and fit the definition of an LC patient [74]. Thus, there is a subgroup of LC patients with significant skin lesions, which could be explained by persistent inflammatory states after acute SARS-Cov-2 infection, even in patients with acute COVID-19 of initially mild symptomatology [75]. Other frequent dermatological lesions in LC are alopecia (56.2%) and “COVID toes” (similar acral lesions), necrotic skin lesions related to the use of vasopressors or decubitus ulcers [76].

## 4. Prospects for the Identification of Skin Lesions Associated with SARS-CoV-2 Infection

The identification, management, and treatment of skin lesions should be known by all levels of health services, both primary care and hospital care. In addition, teams of health care professionals should act in an interdisciplinary manner in the assessment and diagnosis, as well as in the planning and treatment of these skin lesions associated with SARS-CoV-2 infection. In this sense, knowledge of the types of skin manifestations associated with COVID-19, as well as their distribution on the body, prevalence, and affection by age and sex is needed. The causative mechanisms and the treatment of each one of them is essential to develop an appropriate action and care plan personalized according to the type of dermatologic alteration.

To provide high-quality health care to patients suffering from COVID-19-associated skin disorders, a process should be established that could be divided into different stages: (1) Assessment stage, in which the healthcare personnel assesses the patient, carrying out an anamnesis and examination in search of skin lesions; (2) diagnostic stage, which consists of identifying the problems and establishing the care plan. For this, it is essential to know the cutaneous manifestations and their characteristics; (3) therapeutic objectives, in which the interventions to be performed must be established. To establish appropriate health interventions, it is essential to know the physiology and triggering mechanisms of the different types of skin lesions and the treatment proposed for each of them; (4) execution of care, which includes the application of specific treatment for each of the cutaneous manifestations and symptoms associated with COVID-19; (5) disclosure, in which we must consider that we are not only treating a skin lesion but that behind it there is a viral infection (initially unknown), which also must be treated. Therefore, the communication of dermatological lesions and their treatment to the scientific community is essential to establish an adequate evidence-based diagnosis and dermatological care plan, because the prevalence of cutaneous manifestations associated with SARS-CoV-2 will continue to be maintained during the pandemic situation.

## 5. Conclusions

Viral skin infections are some of the most common skin diseases in medical dermatology. To date, the outbreak of COVID-19 has changed the way of life throughout the world, and therefore, also the future of medicine. Scientists have attempted to study all aspects of this viral infection, trying to identify the specific characteristics of SARS-CoV-2 to improve diagnosis and treatment to increase survival. Dermatological science became involved early on, due to the potential of SARS-CoV-2 to cause skin lesions. However, much is still unknown about COVID-19 and even more about the dermatological manifestations observed in patients infected with SARS-CoV-2. Skin alterations are a relevant clinical symptom that broadens the spectrum of diagnostic knowledge about the clinical manifestations associated with COVID-19. The patterns of skin lesions described could serve for the early diagnosis of SARS-CoV-2 infection. It could also broaden the spectrum of differential diagnosis between viral co-infections or adverse reactions to drugs used for COVID-19. To date, the cutaneous manifestations of COVID-19, despite not having a certain predictive value on the stratification of disease severity, can provide data that support the suspicion of an increased risk of associated complications. In some patients, the diagnosis of skin lesions could help in taking measures to prevent infection in patients with minor or asymptomatic symptoms who present skin manifestations associated with COVID-19 without a diagnosis by laboratory tests. Early diagnosis of skin changes could provide adequate treatment to prevent progression to more severe stages of COVID-19. Treatments for the described skin lesions mostly include therapy with anticoagulants, corticosteroids, and antihistamines. Diagnosis of the cutaneous manifestations associated with SARS-CoV-2 infection and their understanding by health care professionals is essential to develop an appropriate plan of care.

## Figures and Tables

**Table 1:** Location and classification of clinical manifestations in patients infected with SARS-CoV-2.

Location	Clinical Manifestations
Respiratory system	Cough, dyspnea, pneumonia, bilateral interstitial inflammatory involvement, acute respiratory distress syndrome, shortness of breath, nasal discharge
Central nervous system	Acute stroke, meningitis, encephalitis, headaches and dizziness, ataxia
Peripheral nervous system	Hypoageusia, hyposmia/anosmia, neuralgia, Guillain-Barré syndrome, chemosensory dysfunction
Endocrine system	Hyperglycemia, ketoacidosis, adrenal insufficiency, thyrotoxicosis
Circulatory system	Myocarditis, heart failure, acute myocardial infarction, cardiomyopathy, shock, arrhythmias, pulmonary thromboembolism, coagulation disorders
Digestive system	Anorexia, nausea, vomiting, diarrhea, abdominal pain, liver injury, sore throat
Excretory system	Acute renal injury, proteinuria, hematuria
Muscle and bone	Skeletal muscle injury, myalgias, generalized weakness, fatigue, arthralgias, decreased bone mineral density
Immune system	Fever, lymphopenia
Lymphatic system	Mediastinal lymphadenopathy
Reproductive system	Orchitis, scrotal discomfort, scrotal pain
Integumentary system	Vesicular rashes, maculopapular rashes, urticarial rashes, petechiae/purpura, acral lesions, liveoid lesions

**Table 2:** Triggering mechanism and treatment of the most common dermatological alterations by SARS-CoV-2.

Dermatological Alteration		Pathophysiology	Treatment	Severity of COVID-19
Vesicular eruptions		Cytokine storm. Direct cytopathic effect of SARS-CoV-2	Expectant management	Mild/moderate
Petechiae/purpura		Catastrophic microvascular injury mediated by complement activation. Cytokine storm. Thrombocytopenia	Topical corticosteroids in mild cases. Systemic corticosteroids in more severe or generalized involvement	Mild/moderate
Acral lesions	Lesions resembling chilblains	Widespread endothelial infection by SARS-CoV-2, endothelial damage, and thrombosis. Overproduction of Type I interferon	Topical corticosteroids alone or in combination with topical antibiotics	Mild
	Acral ischemia	Secondary microthrombosis caused by endothelial damage and vascular disorders. Cytokine storm. Disseminated intravascular coagulation	Anticoagulant therapy. Expectant management	Mild/severe
Liveoid lesions		Hypercogulability. Inflammation caused by SARS-CoV-2 binding to vascular endothelium	Anticoagulant therapy. Expectant management	Severe
Urticarial rashes		Direct mast cell degranulation. Direct cytopathic effect of SARS-CoV-2	Antihistamines. Systemic corticosteroids in low doses.	Moderate
Maculopapular rashes		Cytokine storm. Direct cytopathic effect of SARS-CoV-2	Oral antihistamines. Topical corticosteroids. Systemic corticosteroids in more severe or generalized affections.	Moderate
Vesicular eruptions		Cytokine storm. Direct cytopathic effect of SARS-CoV-2	Expectant management	Mild/moderate
Petechiae/purpura		Catastrophic microvascular injury mediated by complement activation. Cytokine storm. Thrombocytopenia	Topical corticosteroids in mild cases. Systemic corticosteroids in more severe or generalized involvement	Mild/moderate
Acral lesions	Lesions resembling chilblains	Widespread endothelial infection by SARS-CoV-2, endothelial damage, and thrombosis. Overproduction of Type I interferon	Topical corticosteroids alone or in combination with topical antibiotics	Mild
	Acral ischemia	Secondary microthrombosis caused by endothelial damage and vascular disorders. Cytokine storm. Disseminated intravascular coagulation	Anticoagulant therapy. Expectant management	Mild/severe
Liveoid lesions		Hypercoagulability. Inflammation caused by SARS-CoV-2 binding to vascular endothelium	Anticoagulant therapy. Expectant management	Severe
Urticarial rashes		Direct mast cell degranulation. Direct cytopathic effect of SARS-CoV-2	Antihistamines. Systemic corticosteroids in low doses.	Moderate
Maculopapular rashes		Cytokine storm. Direct cytopathic effect of SARS-CoV-2	Oral antihistamines. Topical corticosteroids. Systemic corticosteroids in more severe or generalized affections.	Moderate

## Data Availability

Not applicable.
